# Declining grouper spawning aggregations in Western Province, Solomon Islands, signal the need for a modified management approach

**DOI:** 10.1371/journal.pone.0230485

**Published:** 2020-03-25

**Authors:** A. T. Hughes, R. J. Hamilton, J. H. Choat, K. L. Rhodes

**Affiliations:** 1 Wildlife Conservation Society, Munda, Western Province, Solomon Islands; 2 The Nature Conservancy, Asia-Pacific Resource Center, South Brisbane, Queensland, Australia; 3 ARC Centre of Excellence for Coral Reef Studies, James Cook University, Townsville, Queensland, Australia; 4 College of Science and Engineering, James Cook University, Townsville, Queensland, Australia; 5 MarAlliance, Grass Valley, California, United States of America; Department of Agriculture, Water and the Environment, AUSTRALIA

## Abstract

Globally, groupers (Epinephelidae) that form fish spawning aggregations (FSAs) are highly vulnerable to overfishing and often require site-specific approaches to management. Over 5-years (2009–2013), we conducted underwater visual censuses (UVC) at a well-known spawning site at Njari Island, Gizo, Western Province, Solomon Islands, that supports aggregations of squaretail coralgrouper (*Plectropomus areolatus*), camouflage grouper (*Epinephelus polyphekadion)* and brown-marbled grouper (*E*. *fuscoguttatus*). Findings show that while there were species-specific variations in the duration and timing of the spawning season, aggregation densities peaked from March to June, representing the main spawning season for all three species. For *P*. *areolatus*, gonad analysis from samples taken from 2008 to 2011 confirmed reproductive activity in support of density trends observed through UVC. Over the 5-year UVC monitoring period, FSA densities declined for *P*. *areolatus* and *E*. *polyphekadion*. Conversely, following the first year of monitoring, *E*. *fuscoguttatus* densities increased. These inter-specific differences may reflect variable responses to fishing as shown elsewhere, or for example, differences in recruitment success. In response to known declines in FSAs of these species, in 2018 the Solomon Islands government placed a nationwide ban on these species’ harvest and sale between October and January. As this study shows, this ban does not encompass the peak aggregation period at Njari and will offer limited protection to other FSAs of these species that are known to vary in reproductive seasonality across the Solomon Islands. A more biologically meaningful and practical management strategy would be to implement a nationwide ban on the harvest and sale of these groupers each month between full and new moons when these FSAs form consistently throughout the country. Since effective management of FSAs typically requires a combined approach, spatial management that protects both spawning sites and reproductive migratory corridors is warranted.

## Introduction

Coral reef fisheries provide a valuable source of protein and income for coastal communities throughout the tropics and subtropics, yet these fisheries have come under increasing threat from overfishing, commercialization, habitat loss, population growth and climate change, among other impacts [1–4]. In the tropical Pacific, overfishing is being exacerbated by unsustainable and non-selective use of certain gears, e.g. nighttime spearfishing [5], gillnets and muro-ami, under-valuation of marine resources, enforcement and management shortcomings, limited livelihood alternatives, and excessive targeting of juveniles and (fish) spawning aggregations (FSAs) [6]. For many coral reef fishes, such as groupers (Epinephilidae), reproduction occurs through the formation of FSAs, whereby fish travel varying distances from home reefs and congregate at predictable sites and times over periods typically lasting a few days [7]. These events create an attractive target for both large- and small-scale fisheries, owing to the potential for high catch rates and volumes [6, 8–9].

Globally, there are numerous examples of decreases in spawning populations due to excessive targeting and catch at FSAs [9, 10–14]. Heavy fishing on the FSAs of some species, such as the Critically Endangered (CR) Nassau grouper (*Epinephelus striatus*) [15], has led to several aggregations becoming economically extinct, with other historical aggregations fully extirpated [10, 16–17]. Globally, these declines have come from a combination of small-scale, large-scale and subsistence fishing [13, 18–23], including the Southeast-Asia live reef food fish trade that target FSAs throughout the Indo-Pacific [17].

In the Central and Western Pacific, three grouper species [brown-marbled grouper, *Epinephelus fuscoguttatus* (Forsskål, 1775); camouflage grouper, *E*. *polyphekadion* (Bleeker, 1849); squaretail coralgrouper, *Plectropomus areolatus* (Rüppell, 1830)] commonly form multi-species FSAs that overlap temporally during at least a portion of their respective spawning seasons and in areas proximate to each other [13, 18–19, 24–25]. The timing and location of most FSAs is common knowledge among fishers who have traditionally depended on them for subsistence and, more recently, small-scale commercial interests, including for domestic export [23, 26–27]. In many countries, continuing population growth and an expanding cash economy has intensified FSA fishing [18, 27–29], placing aggregations of these and other species under increasing threat [18, 21, 25]. Indeed, a recent re-examination of extinction threat for epinephelids by the IUCN (International Union for Conservation of Nature) Species Survival Commission Specialist Group for Grouper and Wrasse identified all three species as Vulnerable (VU), largely as a result of FSA fishing [*E*. *polyphekadion* (VU A2bd), *E*. *fuscoguttatus* (VU A2bd+4bd) and *P*. *areolatus* (VU A2bd) [30–32] (www.iucnredlist.org).

In addition to FSA formation, other intrinsic life-history characteristics contribute to their vulnerability, including having short spawning seasonality (e.g. *E*. *polyphekadion*) [33], late maturity [34–35] and competitiveness for bait [28]. For *P*. *areolatus* and *E*. *polyphekadion*, nighttime dormancy in shallow water also increases fishing vulnerability, particularly from spearfishing. Among a number of FSA-forming species, movement to and from spawning sites occurs along common reproductive migratory corridors [29, 36] where fish and fishing are often concentrated. Finally, some species delay spawning until the final day or days once at the site, such that targeted FSA fishing can greatly impact the species’ annual reproductive output [35].

To assist in the design of workable management regimes, an increasing body of work has examined the temporal and spatial dynamics of aggregations and the impacts of fishing [14, 18–19, 23, 25, 28–30, 36–37]. In the Solomon Islands, an abundance of anecdotal information exists on FSA seasonality, lunar periodicity, and the impacts from FSA fishing, but there is only one (peer-reviewed) published account of *E*. *fuscoguttatus*, *E*. *polyphekadion* and *P*. *areolatus* FSAs in the country [18].

The objectives of the current study were to: (1) summarize the temporal aggregation patterns of *P*. *areolatus*, *E*. *fuscoguttatus* and *E*. *polyphekadion* at a multi-species grouper FSA in Western Province, Solomon Islands, by detailing reproductive information on a daily, lunar and seasonal basis; (2) examine changes in the gonadosomatic index of *P*. *areolatus* to confirm that aggregation patterns matched actual reproductive times; and (3) record trends in aggregation abundance (as density) over the course of the 5-year study to identify potential changes. It is envisaged that the findings will be used to inform local communities and government decision makers on both the need for FSA management and to aid in the development of an effective national management strategy for the Solomon Islands.

## Methods

### Study location

**Fig 1:**
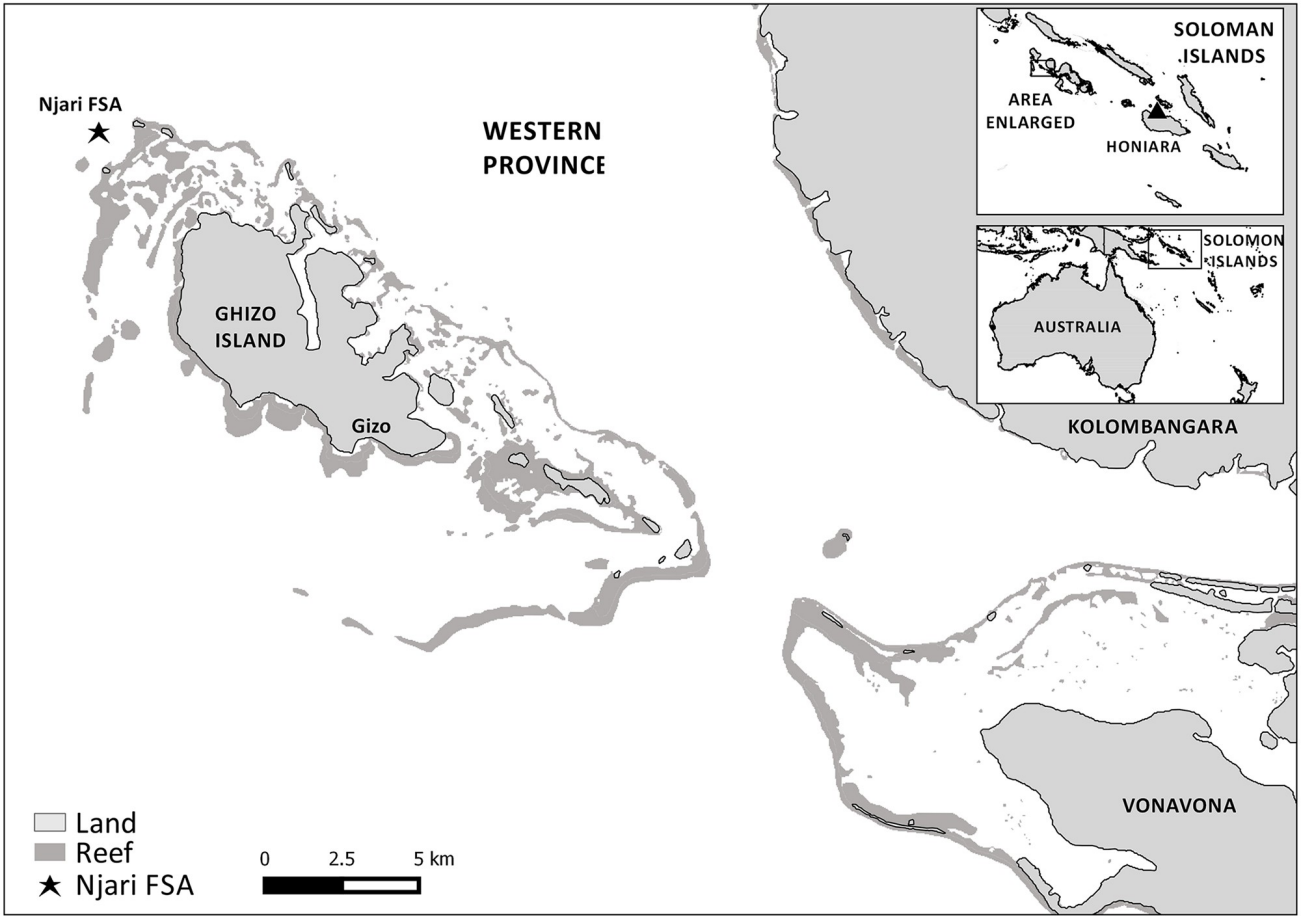
Map of the study site. Map of the study site relative to its regional surroundings. Marketed samples of squaretail coralgrouper *P*. *areolatus* taken for reproductive analysis were derived from Gizo markets, while samples taken by (and subsequently purchased from) local spearfishers were taken from the Njari FSA, located at the northwest extreme of Ghizo Island.

The current study was conducted at Njari Island (8°5 S, 156°49 E), an unprotected multi-species FSA site located within the Ghizo reef system of Western Province, Solomon Islands (Fig 1). The Njari FSA site is part of a large, complex reef (8642 ha) and lagoon (3588 ha) system made up of a mosaic of patch reefs, seagrass and mangrove habitats (Fig 1). The site is located on the seaward edge of a reef promontory on the northwestern tip of the barrier reef system, where *P*. *areolatus*, *E*. *polyphekadion* and *E*. *fuscoguttatus* form overlapping spawning aggregations, similar to other sites in the region. The Njari FSA is located next to one of seven channels within the Ghizo system with daily tides and high levels of water movement characteristic of the site. Njari is well known to local communities and has been frequented by subsistence and artisanal fishers from Ghizo and surrounding islands for at least the past thirty years. Thus, we do not anticipate additional pressure on the site through the publication of the results presented herein.

### The fishery

The Ghizo reef system plays an important role in the supply of fresh protein and income to surrounding coastal communities and supports an active commercial fishery [38] that includes domestic export of reef fish to the capital, Honiara [39]. The town of Gizo fish market is the largest of its kind in Western Province, with reef fish species making up the majority of catch sold locally [38, 40–41]. As with many regional fish markets in the Pacific, including the Solomon Islands, marketed catch is in part supplied through FSA fishing [18, 42]. The Ghizo reef system supports FSAs of a range of species, including groupers. In Gizo, FSA fishing is evident through sudden increases of certain known aggregation spawners at the local fish market during specific lunar phases (Hughes pers. observ.). In Ghizo and neighboring islands, fish harvesting is primarily conducted by nighttime spearfishing, with lesser instances of daytime spearfishing, net and handline fishing [38].

#### Underwater monitoring of FSAs

Exploratory dives were conducted in April and May 2008 to confirm depth profiles for *P*. *areolatus*, *E*. *polyphekadion* and *E*. *fuscoguttatus* FSAs to design a sampling strategy. Preliminary investigations showed that *P*. *areolatus* primarily aggregates within 5–15 m depth, while *E*. *polyphekadion* and *E*. *fuscoguttatus* generally occur between 15–40 m. The combined aggregation area stretches over approximately 250 m of lateral reef area and from 5 to at least 40 m depth. Following these initial investigations, underwater visual census (UVC) (as fish counts) was conducted at the Njari FSA over a 5-year period. Within this period monthly surveys were conducted beginning in April 2009 and lasting until June 2011. Information collected from this 26-month period identified trends in aggregation formation including periods of increased density. Subsequently, UVC surveys were streamlined to focus on the months and days of peak density. All UVC monitoring at the site (2009–2013) utilized two fixed belt transects running the length of the FSAs: a 150-m by 20-m (3000 m^2^) transect at 10-m depth and a second 250-m by 20-m (5000 m^2^) transect installed at 25-m depth. The outer transect boundaries were delimited by steel rebars installed at 25-m intervals to aid in accuracy. A dive pair conducted all UVCs, with Diver 1 recording *P*. *areolatus* and Diver 2 recording *E*. *fuscoguttatus* and *E*. *polyphekadion* along both transects sequentially.

In order to identify daily changes in aggregation density relative to the lunar cycle, fish counts were initiated in April 2009 on the day of full moon and continued until 2 days after new moon (DANM) (18-day period) (S1 Table). Confirmation of the observed daily patterns was performed in June and July 2009 during 10 and 5-day periods, respectively. Counts in June 2009 were taken starting 7 days before new moon (DBFM) until 2 DANM, while those in July 2009 were taken starting 3 DBFM until 2 DANM. Using these data the remaining monitoring protocol was established, which entailed a minimum of two sampling days from 3 to 1 DBNM (S1 Table) in order to gather comparable density data across years and to reduce budgetary and logistical requirements.

For the analysis, prior testing for normality was performed using Shapiro-Wilk test of normality, with density comparisons made using a Kruskal-Wallis H test. All post-hoc analyses used a Dunn’s test of multiple comparisons. Depth-specific and annual density comparisons used data taken between March and June during Days 3, 2 and 1 DBNM. Data were constrained to the months of primary aggregation formation to limit the effects of months where fish were present in low numbers (as during non-reproductive periods) or absent from the site to improve the robustness of the results. Similarly, analyses constrained the days used to periods of the highest observed density (3, 2, 1 DBNM). Annual density comparisons also constrained data to these periods, but combined monthly (and daily) counts within each year regardless of depth. The number of sample days and abundance data by month and year, and by individual transects are provided in (S2 Table).

#### Sub-surface sea temperature monitoring

To determine if FSA formation correlated with changing seasonal water temperature, sub-surface temperature profiles were recorded over 21 consecutive months at Njari (Jan. 2008 –Sept 2009). During these periods, a HOBO Pro V2 temperature logger (Onset Computers, Bourne Massachusetts, USA) was moored at *c*. 20 m depth at the Njari aggregation site. From these data, monthly means were determined. Where two years of data were available (Jan–Sept in each year), a mean of the individual monthly means was calculated.

#### Reproduction

Monthly gonad samples of *P*. *areolatus* were collected either by accompanying night spearfishers exploiting the Njari FSAs or from the Gizo market (Fig 1) from April 2008 to May 2011. Other species were not collected owing to budgetary and logistical constraints. Although the exact origin of marketed samples was at times unknown, reef fish sold at the Gizo market are captured within the Ghizo reef system [38]. Each fish sampled was weighed whole (nearest 1.0 g) before extracting the gonads. Gonads were weighed to the nearest 0.1 g prior to preservation and storage in 4% formaldehyde, 5% acetic acid, and 1.3% calcium chloride. To identify periods of reproductive development and spawning, gonad and body weights were used to calculate the gonadosomatic index (GSI) with the following equation:

$$GSI=gonad\;weight\;body\;weight^{-1}*100$$

Mean sex-specific GSI values were then used to identify monthly trends relative to aggregation formation using both marketed fish and fish taken directly from the Njari FSA. As the fishery operates strictly within the Ghizo reef system, combining samples was considered best in defining the reproductive seasonality of the species. GSI values taken over a 15-d period in April 2009 were used to identify potential spawning times within a calendar month relative to new moon. Histological sectioning of gonads was performed to determine sex, using criteria applied to these species in earlier research (S3 Table) [33, 43]. Sex determinations were coupled with GSI for confirmation of reproductive development and spawning, but otherwise the details of histological investigations are not presented herein.

Gonad samples were obtained through retail purchase of fish obtained either from spearfishers operating at Njari or from the commercial fish market located in Gizo Town. All fish were obtained opportunistically, i.e. when fish were commercially available. The research did not involve any live animals or endangered or protected species. Similar to most developing island nations, the Solomon Islands Government, including MFMR, has no formal committee for reviewing or approving animal handling procedures. The research presented and reported herein conforms to the guidelines for research ethics outlined in the Australian Code for the Care and Use of Animals for Scientific Purposes [44], and the Animal Care and Protection Regulation 2012 (formerly Animal Care and Protection Act 2001 [45]. The research methodology received clearance from the James Cook University Experimentation Ethics Review Committee (Approval Number A1711). Prior to the study, we provided the project overview to the Solomon Islands Ministry of Fisheries and Marine Resources (MFMR), the entity responsible for the Solomon Islands marine resource management, and to the private land and reef owner of Njari Island. Both gave verbal approval for the research.

## Results

### FSA seasonality, temperature, and depth distribution

**Fig 2:**
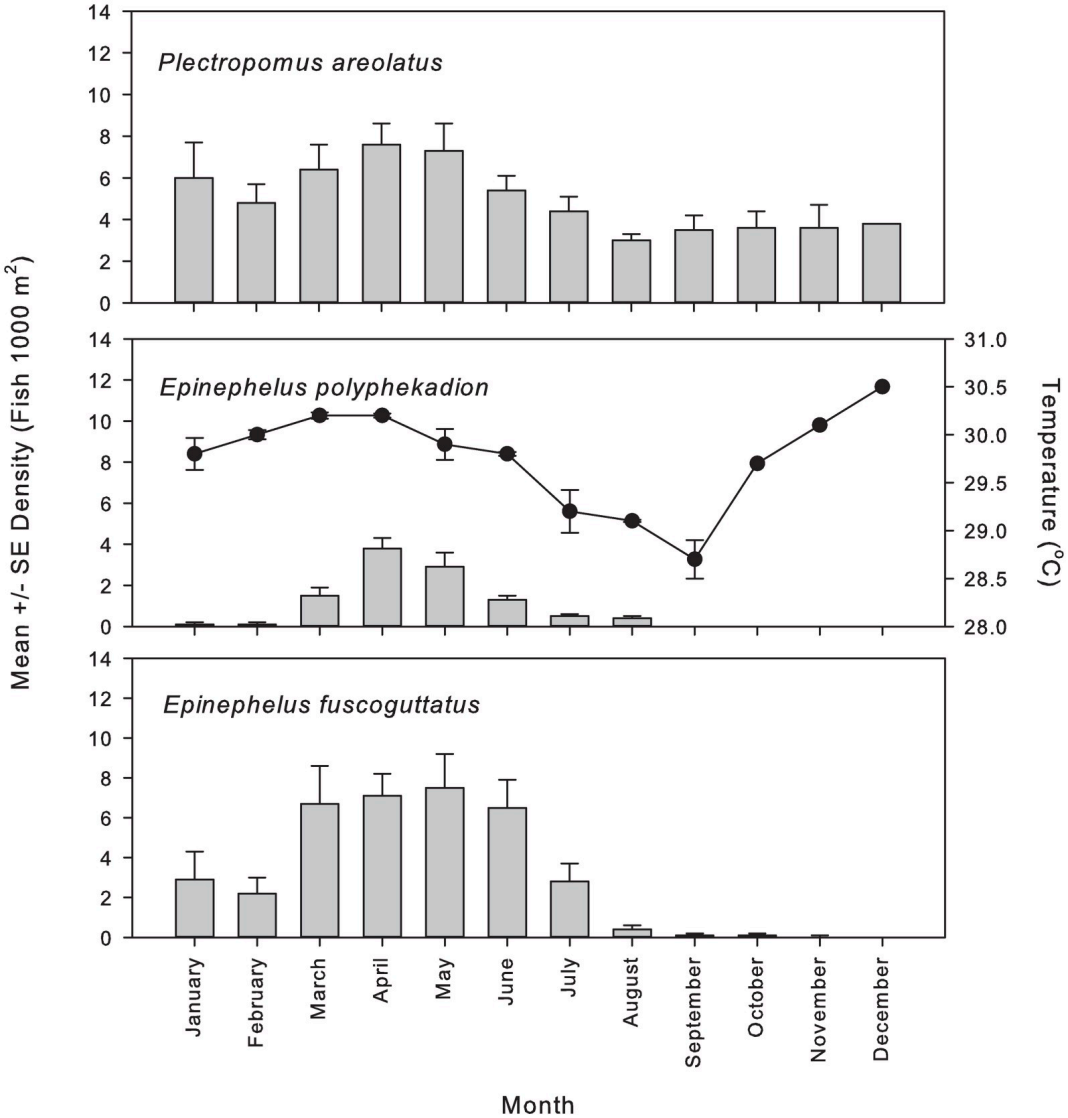
Mean monthly grouper density and temperature at the Njari FSA. Mean ± SE densities for (a) squaretail coralgrouper *P*. *areolatus*, camouflage grouper *E*. *polyphekadion* and brown-marbled grouper *E*. *fuscoguttatus* taken in 2009–2013 are shown against subsurface seawater temperature (°C), with peak fish densities corresponding to winter peaks in temperature. Sub-surface seawater temperatures (b) were taken at 20 m depth over 21 consecutive months in 2008 and 2009, with two years of data available for January to September only.

The seasonal grouper aggregation patterns at Njari differed among the three species. Based on monthly surveys taken between April 2009 and June 2011. *E*. *polyphekadion* formed aggregations between March and August, with a peak in April and May and at higher relative densities from March until June. Among the three species, *E*. *polyphekadion* had the lowest densities overall (Fig 2). *E*. *fuscoguttatus* was present throughout most of the year, but formed substantial FSAs from January to July. Similar to *E*. *polyphekadion*, peaks in *E*. *fuscoguttatus* density occurred in April and May. In contrast, *P*. *areolatus* formed aggregations monthly year-round, with elevated densities from January through June that correspond to the peak spawning season for this species. April was the month of highest mean density for *E*. *polyphekadion* and *P*. *areolatus*, while the highest density of *E*. *fuscoguttatus* was observed in May.

During the peak aggregation months for all three species, sub-surface water temperate ranged between 28.5–30.5 °C. Two periods of elevated water temperature were observed, with the winter peak in temperature coinciding with increasing and peak densities among all three species (Fig 2).

**Fig 3:**
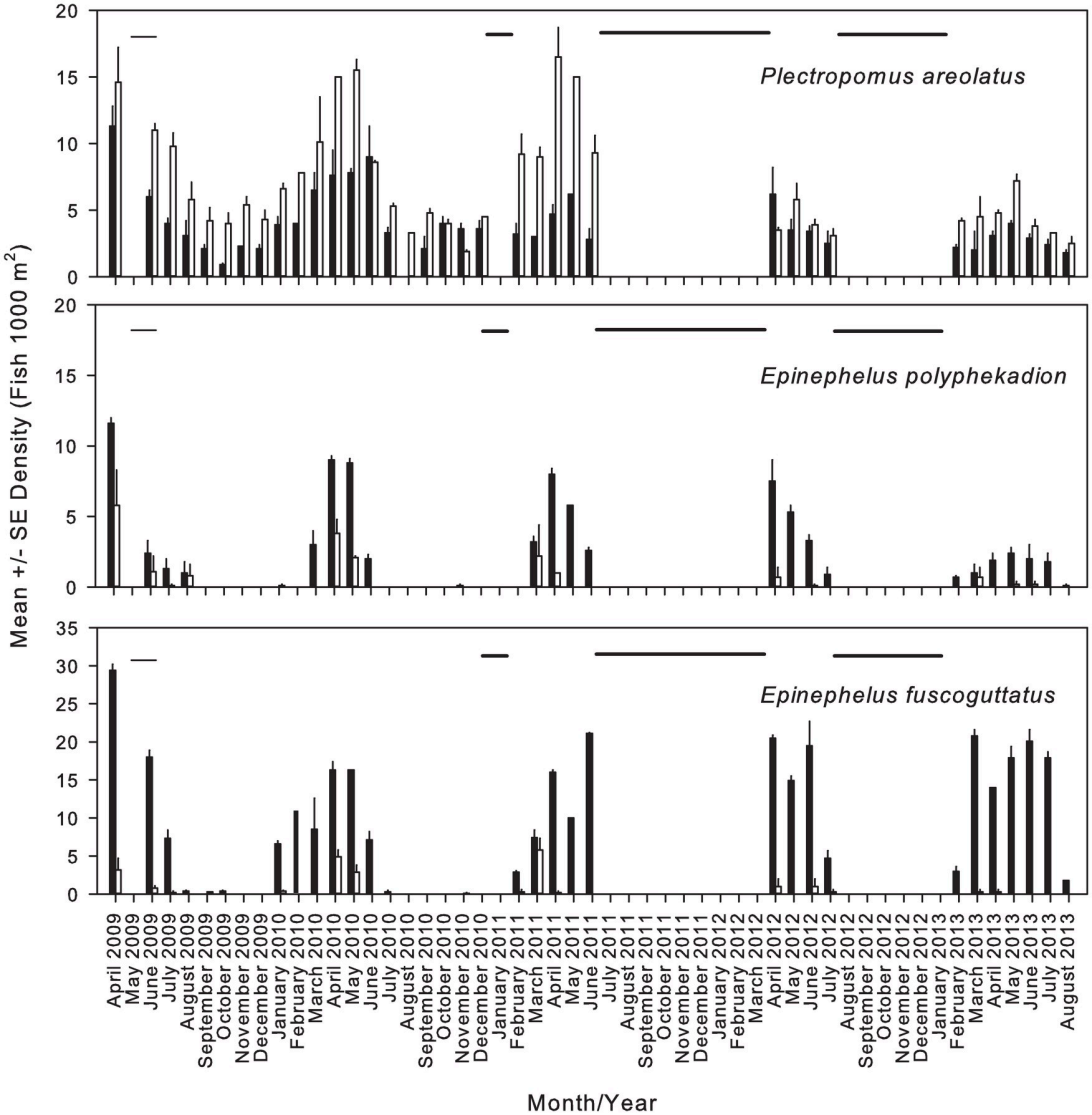
Mean monthly depth- and species-specific density. Monthly patterns of mean ± SE density for squaretail coralgrouper *P*. *areolatus*, camouflage grouper *E*. *polyphekadion*, and brown-marbled grouper *E*. *fuscoguttatus* over a 5-year period. Note that the scale on Y-axis differs among species. Deep transect = black bars; Shallow transects = white bars. Zero-values (horizontal bars) represent months where no UVC was performed (May 2009, January 2011, July 2011—March 2012 and August 2012 –January 2013).

Significant species-specific differences in density were observed between deep and shallow transects for all three species, based on samples taken during dives 3, 2 and 1 DBNM (N = 109) across the 5-yr monitoring period (S2 Table). *P*. *areolatus* densities were significantly greater in the shallow transect (Kruskal-Wallis: H = 57.73, P<0.001) (Fig 3a), while both *E*. *polyphekadion* (Fig 3b) and *E*. *fuscoguttatus* (Fig 3c) aggregated at significantly higher densities within the deeper transect (Kruskal-Wallis: H = 25.19, P <0.001; H = 62.36, P <0.001, respectively).

### Changes in daily aggregation densities between full and new moon

**Fig 4:**
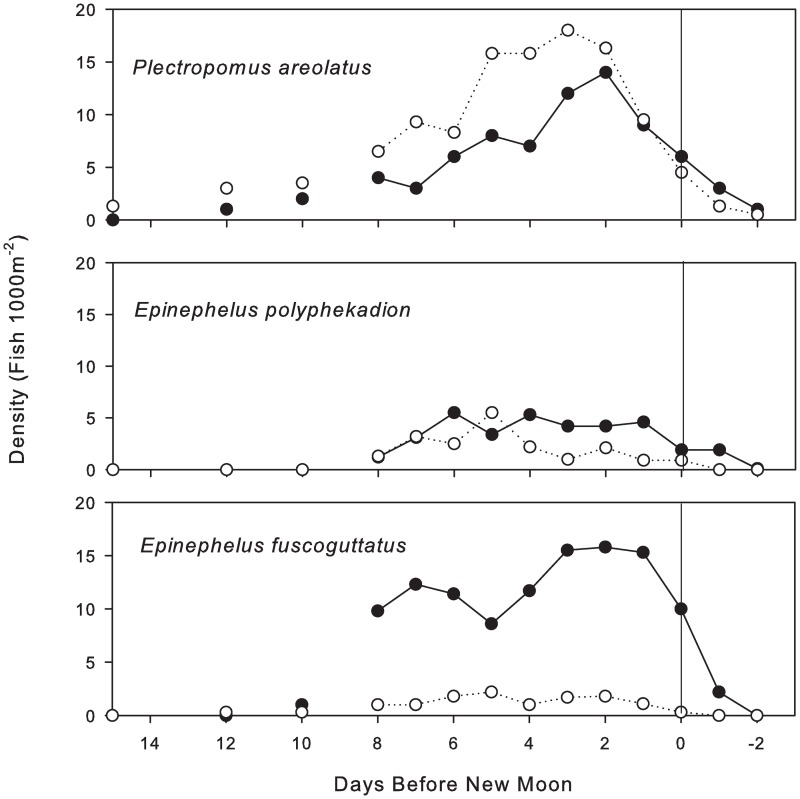
Fish density at the Njari FSA by lunar day. Depth-specific fish densities relative to the day before new moon for UVC estimates of squaretail coralgrouper *P*. *areolatus*, camouflage grouper *E*. *polyphekadion*, and brown-marbled grouper *E*. *fuscoguttatus*, at the Njari FSA site in April 2009. Shallow transects = hollow circles; Deep transects = filled circles; vertical line = new moon.

Intensive daily surveys that were conducted during the peak spawning period in April 2009 revealed that densities for all three species at the FSA site gradually increased between the full and new moons over a 15 to 12-d period before new moon (Fig 4). For *P*. *areolatus*, fish arrived at the FSA site up to 15 DBNM (Fig 4a), while both *E*. *polyphekadion* and *E*. *fuscoguttatus* FSA build-up initiated on the 2^nd^ quarter moon 12 DBNM (Fig 4b and 4c, respectively). Peaks in densities were observed for all species from 3 to 1 DBNM. Following new moon, a rapid decline was observed among all species to indicate that spawning had concluded. By 2 DANM, densities for all three species were reduced to those observed during non-reproductive periods.

### Inter-annual changes in aggregation densities

**Fig 5:**
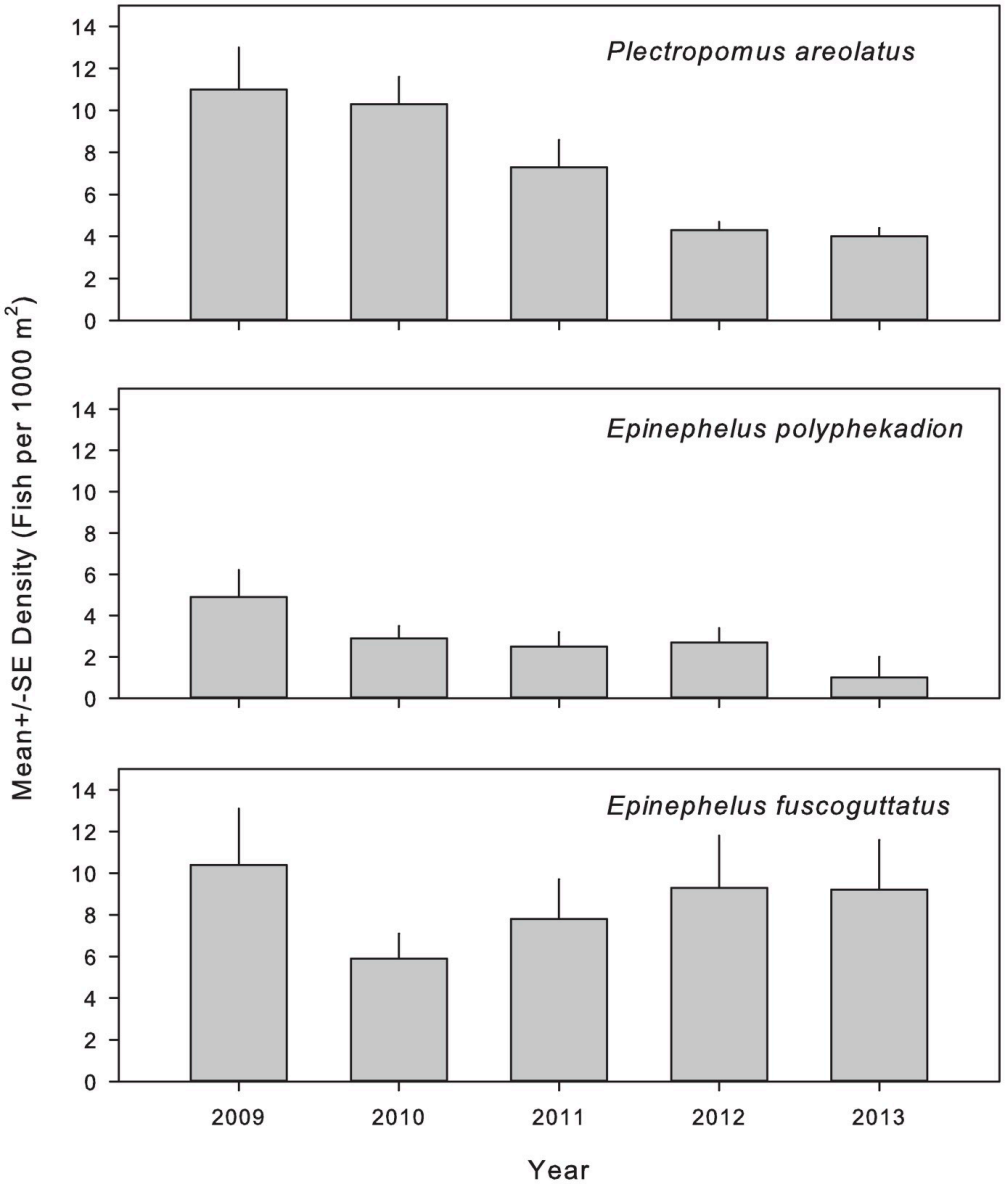
Inter-annual fish density changes at the Njari FSA. Inter-annual changes in mean ± SE density for squaretail coralgrouper *P*. *areolatus* (N = 4238), camouflage grouper *E*. *polyphekadion* (N = 1420) and brown-marbled grouper *E*. *fuscoguttatus* (N = 4495) taken by UVC at the Njari FSA site. Densities reflect combined data taken 3, 2 and 1 DBNM from March to June in all sample years. Significant declines in density were identified for squaretail coralgrouper *P*. *areolatus* only.

Although declines were observed in both *E*. *polyphekadion* and *P*. *areolatus* over the 5-year survey, declines were significant for *P*. *areolatus* only (Kruskal-Wallis: *P*. *areolatus*, H = 27.92, P<0.001; *E*. *polyphekadion*, H = 7.23, P = 0.124) (Fig 5). For *P*. *areolatus*, post-hoc testing showed significant differences (P<0.05) between 2009 and both 2012 (Q = 3.31) and 2013 (Q = 3.52). Similarly, 2010 was shown to be different from 2012 (Q = 3.85) and 2013 (Q = 4.08). No significant changes in density were observed among years for *E*. *fuscoguttatus*, over the 5-year study period (H = 2.04, P = 0.729). In contrast to the other species, densities of *E*. *fuscoguttatus* increased from 2010 until the conclusion of the survey.

### Gonadosomatic index

**Fig 6:**
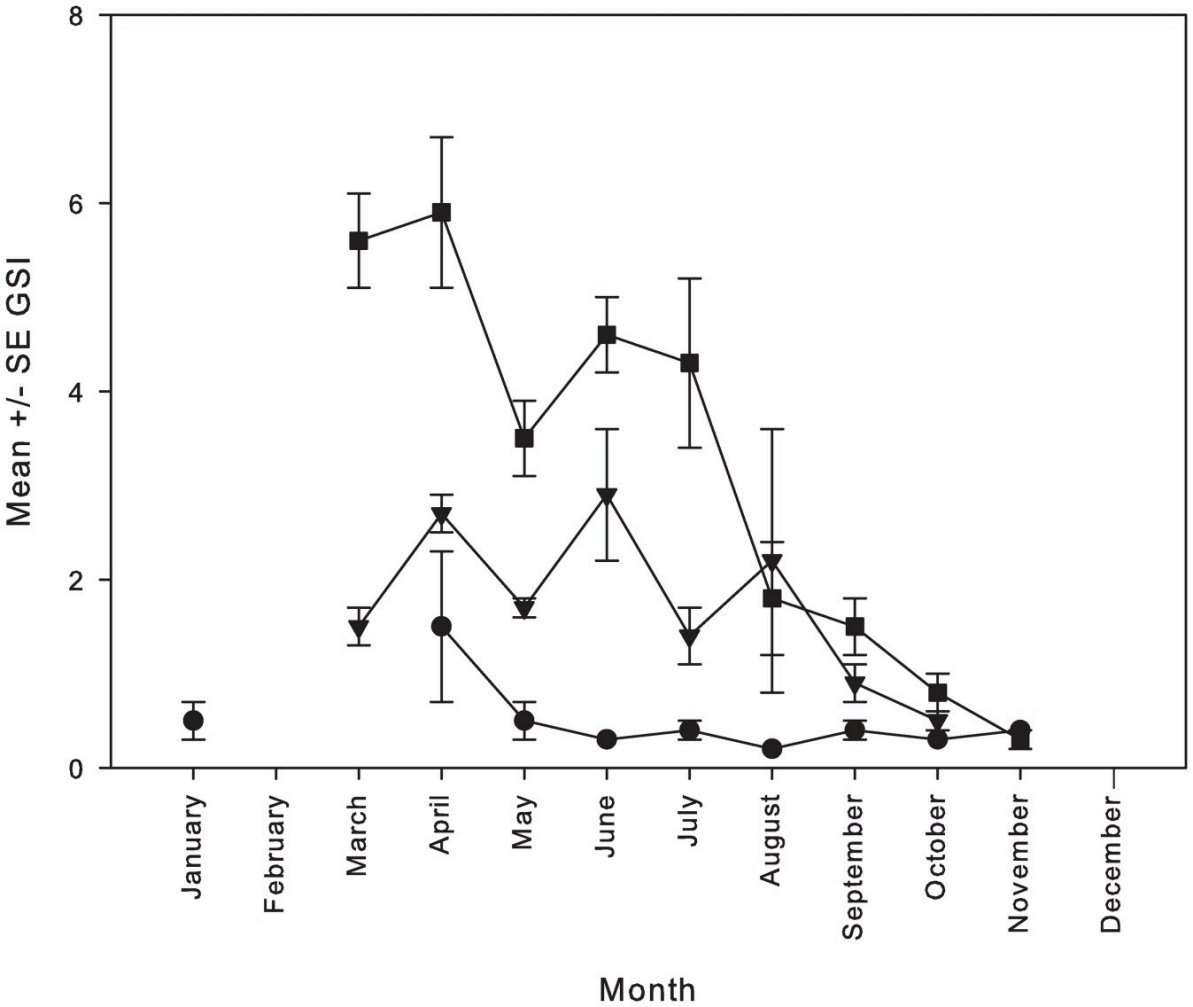
Mean monthly gonadosomatic indices for squaretail coralgrouper. Mean ± SE GSI for sampled squaretail coralgrouper *P*. *areolatus* collected directly from the Njari FSA (n = 247) or from the Gizo market (n = 178). No samples were collected in the months of February or December. Mature females = squares; Mature males = triangles; Immature individuals = circles.

A total of 425 individuals taken from combined FSAs (n = 247) and market-derived (n = 178) sampling were used to establish seasonal reproductive trends in aggregation formation for *P*. *areolatus*. Seasonal GSI values for *P*. *areolatus* largely mirrored changes in density seen at the Njari FSA, with peak values shown in March and April, although sample sizes in some months (e.g. December, January and–February) were either zero or insufficient to establish confident trends in those months (Fig 6; S4 Table). Regardless, seasonal GSI peak values in March and April confirmed the primary reproductive periods. Thereafter, GSI declined and values remained high until at least August, reflective of the protracted spawning period that often characterizes *P*. *areolatus*. Females with late-stage ooctye development (F3-mature) were still evident in September and one of nine females sampled in October was spent, demonstrating that at least some reproductive activity occurs during most months of the year. Elevated GSI values among males mirrored those of females temporally. As expected, GSI values among immature individuals were less than 1% of total body weight throughout the year.

**Fig 7:**
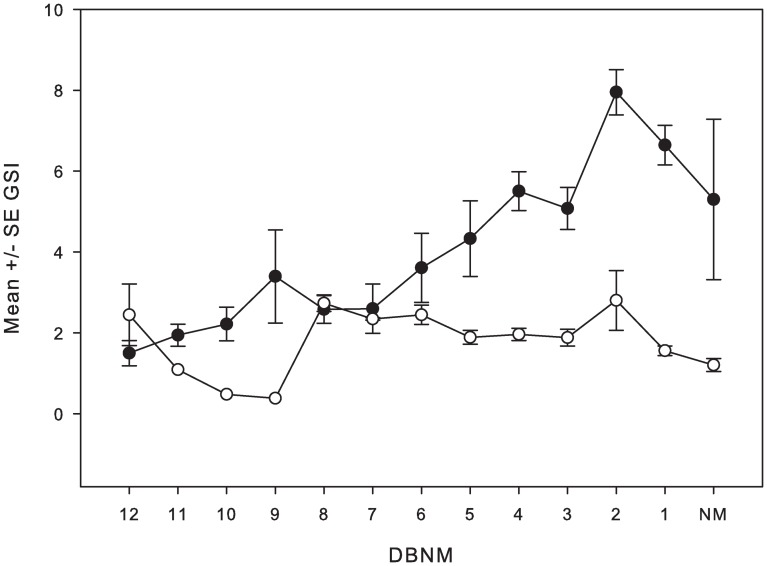
Changes in sex-specific gonadosomatic indices by lunar day for squaretail coralgrouper. Mean ± SE GSI for reproductively active squaretail coralgrouper *P*. *areolatus* females (closed circles) and males (open circles) pooled by lunar day over the 12-day period before new moon (NM). GSI values are from combined marketed and speared samples taken within each sampling month during the study.

Within months, GSI values showed a continual increase for sampled females beginning 12 DBNM until 2 DBNM, when values declined to indicate that at least some spawning was occurring among females during those periods (Fig 7). These latter declines may also suggest a protracted spawning period for the species between 2 DANM and new moon. In contrast, the GSI values among active males remained relatively low throughout the 2-week period.

## Discussion

Temporal and spatial patterns of *E*. *fuscoguttatus*, *E*. *polyphekadion* and *P*. *areolatus* FSAs were examined as part of a 5-year monitoring study to identify reproduction patterns and potential inter-annual changes in aggregation density. Results showed variable species-specific seasonality in aggregation formation and density among the three species, but commonality in lunar periodicity. Specifically, *E*. *polyphekadion* aggregated primarily over a 4-month period, *E*. *fuscoguttatus* over a 6–7 month period and *P*. *areolatus* formed aggregations monthly. Peaks in FSA density were greatest for all three species in April and May, with all species forming FSAs in an approximate two-week period leading up to new moon. For *P*. *areolatus*, increases in GSI mirrored seasonal patterns in peak FSA density, suggesting that most of the annual reproductive output likely occurs during these months (March–July). Gonad samples were minimal or nil in some months, resulting in an incomplete understanding of reproductive seasonality. FSA density peaks from March through May coincided with one of two periods of subsurface seawater temperature highs. In contrast, during periods of summer highs in seawater temperature, FSAs of *E*. *polyphekadion* and *E*. *fuscoguttatus* were absent, suggesting that other individual or combined environmental factors are influencing FSAs formation and reproduction.

The initiation of aggregation formation varied among species within each month that FSAs formed, with *P*. *areolatus* arriving at the site up to 15 DBNM, and FSAs of *E*. *fuscoguttatus* and *E*. *polyphekadion* forming up to 12 DBNM. By 2 DANM, all individuals had dispersed from the FSA site suggesting that spawning had occurred. Although declines in density were observed for both *E*. *polyphekadion* and *P*. *areolatus*, significant declines were only observed for the latter. In contrast, increases in FSA density for *E*. *fuscoguttatus* following 2010 may indicate variable responses among these three species to fishing as shown elsewhere [28]. Alternatively, these differences may reflect species-specific variations in recruitment success, as one example.

At Njari, temporal patterns of FSA formation generally reflected those of other areas studied to date where these three species co-aggregate (Palau [19], Papua New Guinea [25], Roviana, Solomon Islands, [23] and Pohnpei [14]). In all instances, *E*. *polyphekadion* has the shortest spawning season, with *E*. *fuscoguttatus* intermediate and *P*. *areolatus* having the longest. Curiously, *P*. *areolatus* forms monthly FSAs in areas within and adjacent to some locales within the Coral Triangle [23, 25, 46], which contrasts with a shorter seasonal pattern of FSA formation in the central and western Pacific (i.e. 3–5 months [19, 47]; Rhodes et al. *unpublished manuscript*). The only known exception for *P*. *areolatus* in the Coral Triangle is Ayau, Raja Ampat, Indonesia, which appears to have a 5-month spawning season [48], however year-round sampling is needed to confirm this. The driver(s) responsible for these regional variations are currently unknown.

In addition to seasonal variations in spawning times, variations in depth distribution are common among the three species, with *P*. *areolatus* typically forming FSAs in shallower water, while *E*. *fuscoguttatus* FSAs tend to be deep and *E*. *polyphekadion* either intermediate of similar in depth to *E*. *fuscoguttatus* [13, 21, 49]. While this pattern does not always hold [49], species-specific variations in depth distribution are common to a number of sites within the distributional range of these species. Although little has been reported on the nighttime habits of *E*. *fuscoguttatus*, the other two species demonstrate nighttime dormancy and tend to shelter in holes in the reef, where they are often easily accessible to nighttime spearfishing [18]. At Njari, spearfishers have been observed to collect 15 *P*. *areolatus* fisher^-1^ hr^-1^ [42], while other regional reports estimate catch rates between 16 and 20 fish fisher^-1^ hr^-1^ [18, 23]. In Pohnpei, fishers using hook-and-line captured *P*. *areolatus* at rates of 3.8 fish per hr^-1^ [47]. Catch rates from nighttime spearfishing have not been recorded for *E*. *polyphekadion*. However, catch rates estimates for hook-and-line fishing identified an eight-fold higher vulnerability to this gear for *E*. *polyphekadion* than for *E*. *fuscoguttatus* [28], highlighting the variable responses of these species to different gear types. These differences may at least partly explain the variable trends in annual density observed among these fishes at Njari, however there a number of other factors that may be contributing to these differences, among them variable recruitment success.

The result from this study supports observations made elsewhere in the region that have described the tendency for *E*. *fuscoguttatus*, *E*. *polyphekadion* and *P*. *areolatus* to form overlapping FSAs that are both highly attractive to fishers and exceptionally vulnerable to extirpation. In the Solomon Islands, current nationwide measures to protect FSAs through seasonal sales and catch bans from October to January are of limited value, since they only cover a portion on the peak reproductive season in the Western Province and exclude the peak reproductive periods for a number of FSAs, including Njari. In the Solomon Islands, reproductive seasonality for these three species varies widely throughout the country. For example, peak spawning times vary from January through June in Ghizo, November through April in Roviana Lagoon, and February through June in Marovo Lagoon, over a distance as little as 60 km [18, 21]. Recent fisher interviews conducted in eastern Marovo Lagoon (Western Province) identified variations in peak spawning times over distances as small as *c*. 100 km (Hughes *pers*. *observ*.), while in Ontong Java atoll, two peak spawning periods occur during non-overlapping times of the year [50]. Regardless, in each locale, aggregation formation is consistent in that it occurs in the days leading up to new moon. Thus, while the one-size-fits-all national seasonal ban will prove to be ineffective, a lunar ban on sales and capture between the full and new moons has the potential to protect FSAs across the nation. While the widespread and complex geographical nature of the Solomon Islands will make enforcing any ban challenging without community involvement, focusing management efforts on fish retail facilities would simplify enforcement logistics and limit resource usage. This is particularly true for Honiara, which represents the largest consolidator of reef fish being sold in the nation (Solomon Islands Government MFMR, *unpublished data*). In other parts of the Solomon Islands, provincial fisheries officers could provide enforcement assistance where fish markets are centralized, such as Gizo and Munda (Western Province). Spatial closures (locally managed marine areas) that encompass FSAs can provide additional protection where local communities are willing to monitor and enforce them. Given the known dangers of food insecurity in the Solomon Islands [2] and the need for maintaining or expanding existing fish stocks, the protection of FSAs of these and other species is of the utmost importance and should be a central focus of management, as outlined in the national government’s management strategy [51]. While a number of efforts are underway in the Solomon Islands to improve food security through aquaculture, for these and other higher trophic level species, aquaculture is not a viable option. A number of impediments exist for successful aquaculture of these species, among them the technical and monetary requirements needed, which do not currently exist in the Solomon Islands. Moreover, higher trophic level species such as groupers require substantial nutritional inputs [52]. With the inherent food security issues facing the Solomon Islands, providing fishmeal (as an example) to rear medium-to-large bodied carnivores is impractical owing to the need to procure lower trophic level fish species from the wild that would otherwise be available as a direct food source to Solomon Islanders [17, 52]. Other measures, such as TURFs (territorial use rights for fisheries) and rights-based fishing already exist in the Solomon Islands as locally managed marine areas and marine tenureships. These are most effective where village chiefs retain strong enforcement capability, however these arrangements are eroding in many parts of the Solomon Islands and other regional jurisdictions where they exist. Strengthening these arrangements through improved awareness and government and non-government management assistance could add to the protection of these species during aggregation periods. Finally, rights-based fishing using catch quotas or other types of efforts to control or attempt to limit the number or volume of fish taken from FSA is not a viable option for many aggregating species, including groupers, owing to hyperstability in catch, which masks aggregation declines even as fishing efforts remain constant [28, 53, 54]. Such arrangements would require a level of monitoring, including underwater monitoring, that is not typical of community-based monitoring efforts. These various complications highlight the need for practical management measures such as those recommended herein that protect FSA sites and the fish utilizing them during reproductive periods. To do otherwise will likely have dire consequences for these species and for future generations of Solomon Islanders.

## Supporting information

S1 Table Lunar days on which transects were surveyed by month and year. Lunar days on which transects were surveyed during each month that monitoring was conducted at Njari, Western Province, Solomon Islands. Lunar day 0 = New Moon. Ticks represent months where underwater visual census (UVC) was conducted. Shaded cells are periods of peak density for all three species (squaretail coralgrouper, *Plectropomus areolatus*; camouflage grouper *Epinephelus polyphekadion*; brown-marbled grouper *Epinephelus fuscoguttatus*). Monthly and annual density profiles and comparisons represent values taken from 3 days before new moon to 1 day after new moon unless otherwise stated.
(DOCX)

S2 Table Transect- and species-specific sample numbers taken by underwater visual census from 2009 to 2013. Raw abundance data, as sample numbers, of *P*. *areolatus*, *E*. *polyphekadion* and *E*. *fuscoguttatus* taken at deep (A) and shallow (B) water transects over the 5-year survey period (2009–2013).
(DOCX)

S3 Table Macroscopic and microscopic criteria for gonad development stages of individuals. Diagnostic criteria for determinations of sex and gonad development stage for *P*. *areolatus* taken from the Njari FSA and Gizo fish market between April 2008 –March 2011.
(DOCX)

S4 Table Number of monthly squaretail coralgrouper *P*. *areolatus* gonad samples collected. Number of squaretail coralgrouper *P*. *areolatus* gonad samples collected each month from FSA sites and non-FSA (market) sources around the Ghizo reef systems of Western Province, Solomon Islands. Sample numbers represent individuals taken from combined years of sampling (April 2008 –March 2011).
(DOCX)
