# Supplementary material for: Declining grouper spawning aggregations in Western Province, Solomon Islands, signal the need for a modified management approach
Source: PLoS One. 2020 Mar 25;15(3):e0230485. doi: 10.1371/journal.pone.0230485 (PMC7094847; doi:10.1371/journal.pone.0230485)
Supplement: S1 Table — Lunar days on which transects were surveyed during each month that monitoring was conducted at Njari, Western Province, Solomon Islands. Lunar day 0 = New Moon. Ticks represent months where underwater visual census (UVC) was conducted. Shaded cells are periods of peak density for all three species (squaretail coralgrouper, Plectropomus areolatus; camouflage grouper Epinephelus polyphekadion; brown-marbled grouper Epinephelus fuscoguttatus). Monthly and annual density profiles and comparisons represent values taken from 3 days before new moon to 1 day after new moon unless otherwise stated. (DOCX) [file pone.0230485.s001.docx]

**S1 Table. Lunar days on which transects were surveyed by month and year.** Lunar days on which transects were surveyed during each month that monitoring was conducted at Njari, Western Province, Solomon Islands. Lunar day 0 = New Moon. Ticks represent months where underwater visual census (UVC) was conducted. Shaded cells are periods of peak density for all three species (Squaretail coralgrouper, *Plectropomus areolatus*; Camouflage grouper *Epinephelus polyphekadion*; Brown-marbled grouper *Epinephelus fuscoguttatus*).

| Date | Lunar days UVC conducted at FSA | | | | | | | | | | | | | | | | | |
| --- | --- | --- | --- | --- | --- | --- | --- | --- | --- | --- | --- | --- | --- | --- | --- | --- | --- | --- |
|  | 15 | 14 | 13 | 12 | 11 | 10 | 9 | 8 | 7 | 6 | 5 | 4 | 3 | 2 | 1 | 0 | -1 | -2 |
| Apr 09 | ✓ |  |  | ✓ |  | ✓ |  | ✓ | ✓ | ✓ | ✓ | ✓ | ✓ | ✓ | ✓ | ✓ | ✓ | ✓ |
| May 09 |  |  |  |  |  |  |  |  |  |  |  |  |  |  |  |  |  |  |
| Jun 09 |  |  |  |  |  |  |  |  | ✓ | ✓ | ✓ | ✓ |  | ✓ | ✓ | ✓ | ✓ | ✓ |
| Jul 09 |  |  |  |  |  |  |  |  |  |  |  |  | ✓ | ✓ | ✓ | ✓ | ✓ | ✓ |
| Aug 09 |  |  |  |  |  |  |  |  |  |  |  | ✓ | ✓ | ✓ | ✓ | ✓ |  |  |
| Sep 09 |  |  |  |  |  |  |  |  |  |  |  |  | ✓ | ✓ | ✓ | ✓ |  |  |
| Oct 09 |  |  |  |  |  |  |  |  |  |  |  |  |  | ✓ | ✓ |  |  |  |
| Nov 10 |  |  |  |  |  |  |  |  |  |  |  |  | ✓ | ✓ |  |  |  |  |
| Dec 10 |  |  |  |  |  |  |  |  |  |  |  |  | ✓ | ✓ | ✓ | ✓ |  |  |
| Jan 10 |  |  |  |  |  |  |  |  |  |  |  |  |  | ✓ | ✓ |  |  |  |
| Feb 10 |  |  |  |  |  |  |  |  |  |  |  |  |  |  | ✓ | ✓ |  |  |
| Mar 10 |  |  |  |  |  |  |  |  |  |  |  |  | ✓ |  | ✓ | ✓ |  |  |
| Apr 10 |  |  |  |  |  |  |  |  |  |  |  |  |  | ✓ | ✓ | ✓ |  |  |
| May 10 |  |  |  |  |  |  |  |  |  |  |  |  |  | ✓ | ✓ |  |  |  |
| Jun 10 |  |  |  |  |  |  |  |  |  |  |  |  |  | ✓ | ✓ |  |  |  |
| Jul 10 |  |  |  |  |  |  |  |  |  |  |  |  |  | ✓ | ✓ |  |  |  |
| Aug 10 |  |  |  |  |  |  |  |  |  |  |  |  |  |  | ✓ | ✓ |  |  |
| Sep 10 |  |  |  |  |  |  |  |  |  |  |  |  |  | ✓ | ✓ |  |  |  |
| Oct 10 |  |  |  |  |  |  |  |  |  |  |  |  |  | ✓ | ✓ |  |  |  |
| Nov 10 |  |  |  |  |  |  |  |  |  |  |  |  |  | ✓ | ✓ |  |  |  |
| Dec 10 |  |  |  |  |  |  |  |  |  |  |  |  |  | ✓ | ✓ |  |  |  |
| Jan 11 |  |  |  |  |  |  |  |  |  |  |  |  |  |  |  |  |  |  |
| Feb 11 |  |  |  |  |  |  |  |  |  |  |  |  |  | ✓ | ✓ |  |  |  |
| Mar 11 |  |  |  |  |  |  |  |  |  |  |  |  |  | ✓ | ✓ |  |  |  |
| Apr 11 |  |  |  |  |  |  |  |  |  |  |  |  |  | ✓ | ✓ |  |  |  |
| May 11 |  |  |  |  |  |  |  |  |  |  |  |  |  | ✓ | ✓ |  |  |  |
| Jun 11 |  |  |  |  |  |  |  |  |  |  |  |  |  | ✓ | ✓ | ✓ |  |  |
| Apr 12 |  |  |  |  |  |  |  |  |  |  |  |  |  | ✓ | ✓ |  |  |  |
| May 12 |  |  |  |  |  |  |  |  |  |  |  |  |  | ✓ | ✓ |  |  |  |
| Jun 12 |  |  |  |  |  |  |  |  |  |  |  |  | ✓ | ✓ | ✓ |  |  |  |
| Jul 12 |  |  |  |  |  |  |  |  |  |  |  | ✓ | ✓ | ✓ | ✓ |  |  |  |
| Feb 13 |  |  |  |  |  |  |  |  |  |  |  |  | ✓ | ✓ |  |  |  |  |
| Mar 13 |  |  |  |  |  |  |  |  |  |  |  |  | ✓ | ✓ |  |  |  |  |
| Apr 13 |  |  |  |  |  |  |  |  |  |  |  |  | ✓ | ✓ |  |  |  |  |
| May 13 |  |  |  |  |  |  |  |  |  |  |  |  |  | ✓ | ✓ |  |  |  |
| Jun 13 |  |  |  |  |  |  |  |  |  |  |  |  |  | ✓ | ✓ |  |  |  |
| Jul 13 |  |  |  |  |  |  |  |  |  |  |  |  |  | ✓ | ✓ |  |  |  |
| Aug 13 |  |  |  |  |  |  |  |  |  |  |  |  |  | ✓ | ✓ |  |  |  |
