# Supplementary material for: Declining grouper spawning aggregations in Western Province, Solomon Islands, signal the need for a modified management approach
Source: PLoS One. 2020 Mar 25;15(3):e0230485. doi: 10.1371/journal.pone.0230485 (PMC7094847; doi:10.1371/journal.pone.0230485)
Supplement: S2 Table — Raw abundance data, as sample numbers, of P. areolatus, E. polyphekadion and E. fuscoguttatus taken at deep (A) and shallow (B) water transects over the 5-year survey period (2009–2013). (DOCX) [file pone.0230485.s002.docx]

**S2 Table. Transect- and species-specific sample numbers taken by underwater visual census from 2009 to 2013**. Raw abundance data, as sample numbers, of *Plectropomus areolatus*, *Epinephelus polyphekadion* and *Epinephelus fuscoguttatus* taken at deep (A) and shallow (B) water transects over the 5-year survey period (2009-2013).

| Year | Month | Total Sampling Days | *P. areolatus* | | *E. polyphekadion* | | *E. fuscoguttatus* | |
| --- | --- | --- | --- | --- | --- | --- | --- | --- |
|  | | | Transect | | | | | |
|  |  |  | A | B | A | B | A | B |
| 2009 | March | 0 | 0 | 0 | 0 | 0 | 0 | 0 |
|  | April | 3 | 136 | 175 | 139 | 70 | 353 | 38 |
|  | May | 0 | 0 | 0 | 0 | 0 | 0 | 0 |
|  | June | 2 | 48 | 88 | 19 | 9 | 144 | 6 |
|  | *Total* | *5* | *184* | *263* | *158* | *79* | *497* | *44* |
| 2010 | March | 2 | 52 | 81 | 24 | 0 | 68 | 0 |
|  | April | 2 | 61 | 120 | 72 | 30 | 130 | 35 |
|  | May | 2 | 62 | 124 | 70 | 30 | 130 | 23 |
|  | June | 2 | 72 | 69 | 16 | 0 | 57 | 0 |
|  | *Total* | *8* | *247* | *394* | *182* | *60* | *385* | *62* |
| 2011 | March | 2 | 30 | 54 | 32 | 13 | 74 | 35 |
|  | April | 2 | 47 | 99 | 80 | 6 | 160 | 1 |
|  | May | 1 | 31 | 45 | 29 | 0 | 50 | 0 |
|  | June | 2 | 28 | 56 | 26 | 0 | 211 | 0 |
|  | *Total* | *7* | *136* | *254* | *167* | *19* | *495* | *35* |
| 2012 | March | 0 | 0 | 0 | 0 | 0 | 0 | 0 |
|  | April | 2 | 62 | 21 | 75 | 4 | 205 | 6 |
|  | May | 3 | 52 | 52 | 80 | 0 | 224 | 0 |
|  | June | 3 | 51 | 35 | 49 | 1 | 293 |  |
|  | *Total* | *8* | *165* | *108* | *204* | *5* | *722* | *15* |
| 2013 | March | 2 | 20 | 27 | 10 | 2 | 208 | 2 |
|  | April | 2 | 31 | 29 | 19 | 0 | 140 | 2 |
|  | May | 2 | 40 | 43 | 24 | 1 | 179 | 0 |
|  | June | 2 | 29 | 23 | 20 | 1 | 201 | 0 |
|  | *Total* | *8* | *120* | *122* | *73* | *4* | *728* | *4* |
