# Supplementary material for: Declining grouper spawning aggregations in Western Province, Solomon Islands, signal the need for a modified management approach
Source: PLoS One. 2020 Mar 25;15(3):e0230485. doi: 10.1371/journal.pone.0230485 (PMC7094847; doi:10.1371/journal.pone.0230485)
Supplement: S3 Table — Diagnostic criteria for determinations of sex and gonad development stage for P. areolatus taken from the Njari FSA and Gizo fish market between April 2008 –March 2011. (DOCX) [file pone.0230485.s003.docx]

**S3 Table: Macroscopic and microscopic criteria for gonad development stages of individuals.** Diagnostic criteria for determinations of sex and gonad development stage for P. areolatus taken from the Njari FSA and Gizo fish market between April 2008 and March 2011.

| Maturity stage | Macroscopic | Microscopic |
| --- | --- | --- |
| Ovaries |  |  |
| F1 (immature) | Small, strand-like tissue, compact, pink or cream; oocytes indiscernible; indistinguishable from M1 males. | Gonad wall thin; tightly packed previtellogenic Stage 1 (nucleolar) and 2 (perinucleolar) oocytes and gonia; no large muscle bundles present. |
| F2 (developing; mature resting) | Relatively small but rounded, greyish with thickened gonad wall; oocytes indiscernible and small (<0.4 mm); Indistinguishable from M2 prior to cortical alveolus stage when oocytes become discernible. | Stages 1 and 2 and cortical alveolus stage oocytes present; represents individuals that are recovering from spawning as indicated by distinct muscle bundles, thick gonad wall, and an absence of post-ovulatory follicles; includes developing (maturing) and inactive females with actively advancing oocytes. |
| F3 (mature, active) | Large and greyish with transparent gonad wall; large vitellogenic oocytes becoming clearly visible and tightly packed | Mainly vitellogenic (Stage 3) oocytes; gonad wall stretched and thin; Stage 1 and 2 oocytes relatively few; no large-scale atresia or post-ovulatory follicles |
| F4 (mature, ripe) | Ovary large, clear, hydrated oocytes visible through wall; typical of individuals just prior to spawning; egg release possible with application of light abdominal pressure | All stages of oocyte development, but dominated by Stage 4 (hydrated) and late Stage 3; yolk fusion and hydration are extensive |
| F5 (post-spawn) | Ovary flaccid with obvious capillaries; few oocytes visible | Post-ovulatory follicles numerous and large prominent muscle bundles scattered throughout the gonad; the gonad wall is thickened; few Stage 3 and/or 4 oocytes may be present and undergoing atresia |
| Testes |  |  |
| M1 (immature/  inactive) | Indistinguishable from F1 females (see the description of F1) | Gonad filled with varying amounts of stroma, usually the dominant feature; 1o and 2o spermatocytes, and spermatids largely absent (< 1%); sperm sinus may be present; large, vacuous central cavity often present; gonial lobules and brown bodies may be evident; some Stage 1 previtellogenic oocytes present in varying amounts, but not the dominant feature; signs of previous reproductive activity, such as muscle bundles, often evident. |
| M2 (developing) | Gonad greyish in appearance and flaccid; M2 individuals are indistinguishable from F2 until milt becomes evident in the sperm sinus | Gonad largely filled with stroma; 1o and 2o spermatocytes, and spermatids in minor proportions, usually < 10% of the volume; sperm sinus present or not; central cavity small or absent; gonia-filled seminiferous tubules a major feature; gonial lobules and brown bodies may be evident; some Stage 1 previtellogenic oocytes possible in varying amounts, but not the dominant feature; past evidence of spawning, such as thick tunica and sperm sinus present. |
| M3 (mature) | Gonad expanding and becoming rounded, large and greyish; milt may run from macroscopically sectioned gonad and some pockets of milt visible through the tunica | 1o and 2o spermatocytes and early sperm formation, sperm crypts and seminiferous tubules; sperm/spermatids and 2o spermatocytes represent 10- 50% of the volume; sperm sinus present with thick or thickening tunica; Stage 1 and 2 oocytes a inor component |
| M4 (mature, active) | Testes large and white with sperm visible in sinuses; milt release with light abdominal pressure | Seminiferous tubules extensive and filled with sperm; gonad volume > 50% sperm/spermatids and few gonia |
| M5 (post-spawn) | Testes flaccid and bloody; sperm release still possible on application of abdominal pressure | Sperm sinuses and seminiferous tubules largely empty with little, no or atretic sperm; muscle bundles abundant and gonad empty of early stages of spermatogenic tissue; thick tunica and some signs of early atresia of remaining sperm/spermatocytes. |
