# Supplementary material for: Declining grouper spawning aggregations in Western Province, Solomon Islands, signal the need for a modified management approach
Source: PLoS One. 2020 Mar 25;15(3):e0230485. doi: 10.1371/journal.pone.0230485 (PMC7094847; doi:10.1371/journal.pone.0230485)
Supplement: S4 Table — Number of squaretail coralgrouper P. areolatus gonad samples collected each month from FSA sites and non-FSA (market) sources around the Ghizo reef systems of Western Province, Solomon Islands. Sample numbers represent individuals taken from combined years of sampling (April 2008 –March 2011). (DOCX) [file pone.0230485.s004.docx]

**S4 Table. Number of monthly squaretail coralgrouper *Plectropomus areolatus* gonad samples collected.** Number of squaretail coralgrouper *P. areolatus* gonad samples collected each month from FSA sites and non-FSA (market) sources around the Ghizo reef systems of Western Province, Solomon Islands. Sample numbers represent individuals taken from combined years of sampling (April 2008 – March 2011).

| Month | Immature | | Female | | Male | | Total |
| --- | --- | --- | --- | --- | --- | --- | --- |
|  | FSA | Non-FSA | FSA | Non-FSA | FSA | Non-FSA |  |
| Jan | 0 | 4 | 0 | 0 | 0 | 0 | 4 |
| Feb | 0 | 0 | 0 | 0 | 0 | 0 | 0 |
| Mar | 0 | 0 | 39 | 0 | 7 | 0 | 46 |
| Apr | 3 | 0 | 24 | 0 | 8 | 3 | 38 |
| May | 9 | 0 | 47 | 3 | 56 | 6 | 121 |
| Jun | 0 | 16 | 14 | 24 | 8 | 3 | 65 |
| Jul | 0 | 7 | 9 | 8 | 6 | 1 | 31 |
| Aug | 0 | 12 | 1 | 7 | 1 | 4 | 25 |
| Sept | 1 | 16 | 1 | 32 | 0 | 14 | 64 |
| Oct | 1 | 10 | 2 | 7 | 2 | 4 | 26 |
| Nov | 0 | 1 | 0 | 4 | 0 | 0 | 5 |
| Dec | 0 | 0 | 0 | 0 | 0 | 0 | 0 |
| Total | 14 | 66 | 137 | 85 | 88 | 35 | 425 |
